# Does policy grow on trees?

**DOI:** 10.1186/1472-6939-15-87

**Published:** 2014-12-22

**Authors:** Bartha M Knoppers

**Affiliations:** Centre of Genomics and Policy, McGill University, Montreal, H3A 0G1 Canada

**Keywords:** Data sharing, Ethics, Global Alliance, Human rights, Policy

## Abstract

Policymaking is both an art and a science. It is a long process of research, debate and consensus (where possible). The elaboration of the *Framework for Responsible Sharing of Genomic and Health-Related Data* serves as an illustration of this process.

## Correspondence

### Introduction: the many aims of ethics policymaking

Scientists have labs and algorithms, but those of us in the “ELSI” (Ethical, Legal and Social Issues) community have only words. Somehow this translates into a common assumption in the biomedical research and funding communities that policies, procedures, consent forms and the like seem to grow on trees, or at least germinate in our dreams and magically appear at will or when needed. Yet, one would be mistaken in this assumption. Ethics policymaking in the biomedical research sector is quite often as delicate, incremental and grounded in precedent as scientific research conducted in a lab.

The aims of ethics policymaking in the biomedical research sector are many: avoid being legalistic; be context-specific; speak plainly; address the diversity of issues, disciplines and cultures involved; facilitate harmonization, compliance and oversight; include all relevant stakeholders; be aspirational; be practical and yet, at the same time, be principled. To that end, there is no doubt of the need for foundational principles – that is, having a common vision – before discussing the possibility of detailed content. One has but to examine the principles invoked by the HUGO Ethics Committee [1] in its first decade of work to see their influence in prospectively addressing emerging issues.

### A case study: the *framework for responsible sharing of genomic and health-related data*

The development of the *Framework for Responsible Sharing of Genomic and Health-Related Data* [2] also serves as a current case-in-point. Inspired by the mission of the Global Alliance for Genomics and Health (GA4GH) to “accelerate progress in human health by helping to establish a common framework of harmonized approaches to enable effective and responsible sharing of genomic and clinical data …” [3], the elaboration of this *Framework* epitomizes the laborious process of international ethics policymaking.

A unique feature of the *Framework*, which has been developed by the Regulatory and Ethics Working Group of the GA4GH and its diverse partners (P3G-IPAC [4]; IRDiRC [5]; BioSHaRE [6]; H3Africa [7] and ISBER [8], among others), is its reliance on human rights, especially the UN’s *Universal Declaration of Human Rights* [9], for guidance and inspiration. Human rights, precisely because they are universal, are more robust and “actionable” than traditional bioethics principles, which necessarily complement these rights [10]. This Framework is guided by the human rights of privacy, non-discrimination and procedural fairness. But most importantly, the *Framework* has as its foundations: the right of all citizens to benefit from scientific progress; and its applications; the right to scientific freedom; and, the rights of data producers and users to be recognized for their contributions to research, balanced by the rights of those who donate their data. The human rights approach is not just a political move, but a strategy that causes the biomedical research and medical community to re-think and re-vitalize the debate surrounding genomic and clinical data sharing. Human rights in the domain of scientific research have largely lain dormant since their first appearance in the United Nations’ 1948 *Universal Declaration of Human Rights*. Though both these rights were integral to the United Nations’ 1966 *International Covenant of Economic, Social and Cultural Rights* (a legally binding covenant signed and ratified by 162 countries), they have been seemingly forgotten by the international bioethics and legal communities. The *Framework* hopes therefore, to finally make these rights “actionable”. But how exactly was this *Framework* built?

### Lessons learned

The most essential ingredient for what was (and is, with all policymaking) inevitably a long and arduous process of discussion, drafting and re-drafting is the involvement of people working closely together. Even more important than the expertise and experience of individuals is their goodwill and capacity for consensus. In short, egos have no place in this environment of mutual trust and respect. Openness to criticism, comments and discussion of everyone’s favorite “wish list” of definitions, clauses, and so on is the ultimate test of policymaking mettle. Moreover, validation of the content of any policy developed through broad exposure and input strengthens eventual buy-in and use by the scientific community. A year in the making, the Framework underwent ten versions following meetings, postings on the Alliance website and public presentations.

While drafting policy is not quantitative, qualitative, empirical or systematic, it does nevertheless require judgement and an in-depth understanding of past and current approaches and controversies. Precedent matters. It seeks to reconcile universals and particulars, the possible and the hoped-for, as well as acquire legitimacy through use and usefulness over time. With respect to the *Framework*, this meant understanding the issues of data sharing as well as a “rigorous reining in” of unrealistic expectations. Moreover, those involved in either drafting the *Framework* or commenting thereon had to be seasoned international ELSI experts in touch with and grounded in the worlds and reality of patients and research participants, labs, biobanks, funders, and, legal or ethical constraints across cultures. This is a dynamic science at work. Cynicism could and should not hold sway in this drafting mission in spite of the constant need for a reality check.

Policymaking also requires patience. One should not expect any immediate tangible impact. Real-world application and citation of what are often self-regulatory approaches occur over the long term. A self-regulatory approach takes time to enter into the scientific culture since it lacks the imprimatur and legitimacy of policies emanating from recognized international bodies such as UNESCO or WHO. But the impact is eventually felt. It is heartening, for example, to witness the long term impact of the 1996 Bermuda Principles that were drafted by the genomics research community [11]. The self-regulatory approach can in fact be advantageous: the development of the *Framework* directly involved the persons it aims to guide, namely scientists, policymakers, research participants, and patients. Thus, the language and content speak to the reality it hopes to serve and guide. However, cynics could see it as self-serving and possibly akin to a conflict of interest. But whether damned for its absence or criticized for its presence, the *Framework* will hopefully fructify these nascent human rights by their very interpretation in the context of global data sharing.

### Conclusion

To summarize then, no, policy does not grow on trees or in ELSI scholars’ fanciful imaginations, but, to be viable, it needs courage, nurturing and rigor…for there is always the possibility of turbulent weather along the way!

## Authors’ information

Bartha Maria Knoppers, Ph.D. O.C., O.Q., is the Director of the Centre of Genomics and Policy, McGill University, Montreal, Quebec. She holds the Canada Research Chair in Law and Medicine and has been involved in international policy making for 25 years including: HUGO, UNESCO, GA4GH, ICGC and ISCF.

## Abbreviations

BioSHaRE: Biobank Standardisation and Harmonisation for Research Excellence project
ELSI: Ethical, legal and social issues
GA4GH: Global Alliance for Genomics and Health
H3Africa: Human Heredity and Health in Africa (H3Africa) Initiative
HUGO: Human Genome Organisation
IRDiRC: International Rare Disease Research Consortium
ISBER: International Society for Biological and Environmental Repositories
P3G-IPAC: Public Population Project in Genomics and Society-International Policy interoperability and data Access Clearinghouse
UNESCO: United Nations Educational, Scientific and Cultural Organization
WHO: World Health Organization.
